# Mass spectrometry-based quantification of the cellular response to ultraviolet radiation in HeLa cells

**DOI:** 10.1371/journal.pone.0186806

**Published:** 2017-11-20

**Authors:** Hong Xu, Xuanyi Chen, Nanjiao Ying, Meixia Wang, Xiaoli Xu, Rongyi Shi, Yuejin Hua

**Affiliations:** 1 Institute of Nuclear-Agricultural Sciences, Zhejiang University, Hangzhou, China; 2 College of Life Information Science and Instrument Engineering, Hangzhou Dianzi University, Hangzhou, China; 3 Zhejiang Institute of Microbiology, Hangzhou, China; The University of Hong Kong, HONG KONG

## Abstract

Ultraviolet (UV) irradiation is a common form of DNA damage that can cause pyrimidine dimers between DNA, which can cause gene mutations, even double-strand breaks and threaten genome stability. If DNA repair systems default their roles at this stage, the organism can be damaged and result in disease, especially cancer. To better understand the cellular response to this form of damage, we applied highly sensitive mass spectrometry to perform comparative proteomics of phosphorylation in HeLa cells. A total of 4367 phosphorylation sites in 2100 proteins were identified, many of which had not been reported previously. Comprehensive bioinformatics analysis revealed that these proteins were involved in many important biological processes, including signaling, localization and cell cycle regulation. The nuclear pore complex, which is very important for RNA transport, was changed significantly at phosphorylation level, indicating its important role in response to UV-induced cellular stress. Protein–protein interaction network analysis and DNA repair pathways crosstalk were also examined in this study. Proteins involved in base excision repair, nucleotide repair and mismatch repair changed their phosphorylation pattern in response to UV treatment, indicating the complexity of cellular events and the coordination of these pathways. These systematic analyses provided new clues of protein phosphorylation in response to specific DNA damage, which is very important for further investigation. And give macroscopic view on an overall phosphorylation situation under UV radiation.

## 1. Introduction

Sunlight is an indispensable energy source for life on the earth while the ultraviolet (UV)-B and UV-C radiation it contains are detrimental to biological organisms [1]. UV, as a major source of DNA damage, is capable of ionizing molecules and generating chemically reactive radicals, which thereby oxidize macromolecules in cells and cause various types of DNA lesions [2]. Among these lesions, double-strand breaks (DSBs) are the most harmful to genome integrity. Nevertheless, cells possess diverse genome protection pathways that can be activated by DNA damage, known as DNA damage response [3]. Once these pathways begin to work, many related proteins are modified by different enzymes in post translational modifications (PTMs) after translation to execute related functions.

More than 400 different types of PTMs have been identified, including phosphorylation [4], acetylation [5], methylation [6] and ubiquitination [7], all of which play very important roles in almost all cellular processes. Phosphorylation is one of the most important PTMs in eukaryotic cells [8]. It is estimated that more than 30% of all proteins in a cell are phosphorylated during its life span and play important roles in regulation of physiological processes including cell cycle [9,10], cell differentiation [11,12], stress response [13,14], exopolysaccharide production [15,16], coordination of division [17–19], cell envelope and virulence [20–22]. Although much work has been done to identify the phosphorylated proteins involved in different cell states, many of the modified proteins and phosphorylation sites are unknown due to technique issues and dynamic cellular changes. Therefore, to better understand the systemic network of phosphorylation under different circumstances is very necessary. In this study, we systematically analyzed the changes of phosphorylated proteins and the modification sites in HeLa cells before and after UV treatment, and determined the potential connections of these modified proteins with UV-induced DNA damage response and repair, which provided meaningful clues for further investigations.

## 2. Materials and methods

### 2.1. Sample preparation

HeLa cells were maintained in SILAC media and expanded for six doublings. After the 6th doubling, HeLa cells with ^13^C^6^-lysine and ^13^C^615^N^4^-Arginine labeling (“heavy”) were harvested and immediately lysed with 2% SDS lysis buffer. About 20 μg of extracted crude proteins were fractionized by 15% SDS-PAGE followed by Coomassie blue staining. After destaining, proteins on gel slice were performed in-gel digestion followed by mass spectrometry analysis. The first 20 peptides with highest intensity were selected to calculate the ^13^C-lysine labeling efficiency.

The average ^13^C^6^-lysine and ^13^C^615^N^4^-Arginine labeling efficiency was calculated to be 95%.The 95% average labeling efficiency shows that the incorporation of^13^C^6^-lysine and ^13^C^615^N^4^-Arginine in HeLa cells fits the labeling criteria for the subsequent SILAC-based phosphorylation quantitative proteomics.

### 2.2. Cell culture and tryptic digestion

The harvested “heavy” and “light” labeled cells were lysed with 2×NETN buffer (200 mM NaCl, 100 mM Tris-Cl, 2mM EDTA, 1.0% NP-40, pH 7.2) supplemented with 0.5% Triton X-100 on ice for 30 min, respectively. The supernatants were saved after 20,000×g centrifugation for 10 min at 4°C. After measurement of protein concentration in“heavy”or“light”labeled supernatant, equal amount of crude proteins in supernatant were mixed and the crude proteins were precipitated by adding trichloroacetic acid (TCA) with 15% final concentration (v/v) (soluble fraction). After washing twice with -20°C acetone, the proteins pellets were dissolved in 100 mM NH_4_HCO_3_ (pH 8.0) for trypsin digestion.

Remaining cell pellets were dissolved in 8 M urea to extract the chromatin-binding proteins. After measurement of protein concentration, equal amount of chromatin-binding proteins in urea solution were mixed and the proteins were precipitated by adding trichloroacetic acid (TCA) with 15% final concentration (v/v) (nuclear pellet fraction). After washing twice with -20°C acetone, the proteins pellets were dissolved in 100 mM NH_4_HCO_3_ for trypsin digestion.

Trypsin (Promega) was added into protein solution with ratio of trypsin to protein at 1:50 (w/w) for digestion at 37°C for 16 hours. DTT (dithothreitol) was then added to final concentration 10 mM followed by incubation at 56°C for 60 min. After that, iodoacetamine was added to alkylate proteins to final concentration 15 mM followed by incubation at room temperature in dark for 30 min. The alkylation reaction was quenched by 30 mM of cysteine (final concentration) at room temperature for another 30 min. Trypsin was then added again with ratio of trypsin to protein at 1:100 (w/w) for digestion at 37°C for 4 hours to complete the digestion cycle.

### 2.3. Affinity enrichment of phosphorylated peptides

Peptide mixtures were first incubated with 50 μL of IMAC microspheres suspension (10 mg/mL in 80% ACN, 6% TFA) with vibration for 30min. The IMAC microspheres with enriched phosphopeptides were collected by centrifugation at 20,000g for 5 min, and the supernatant was removed. To remove nonspecifically adsorbed peptides, the IMAC microspheres were washed with 100 μL of solution containing 50% ACN, 6% TFA and 200 mM NaCl and followed by washing with 100 μL of solution containing 30% ACN, 0.1% TFA. To elute the enriched phosphopeptides from the IMAC microspheres, 100 μL of NH_3_•H_2_O (10%, v/v) was added, and the enriched phosphopeptides were eluted with vibration 30 min and finally centrifuged at 20,000g for 5 min. The supernatant containing phosphopeptides was collected and lyophilized for LC-MS/MS analysis. Sample preparation for massspectrometry.

### 2.4. LC-MS/MS analysis

Peptides were dissolved in solvent A (0.1% FA in 2% ACN), directly loaded onto a reversed-phase pre-column (Acclaim PepMap 100, Thermo Scientific). Peptide separation was performed using a reversed-phase analytical column (Acclaim PepMapRSLC, Thermo Scientific) with a linear gradient of 5–25% solvent B (0.1% FA in 98% ACN) for 50 min, 25–35% solvent B for 10 min, and 35–80% solvent B for 10 min at a constant flow rate of 300 nl/min on an EASY-nLC 1000 UPLC system. The resulting peptides were analyzed by Q Exactive TMPlus hybrid quadrupole-Orbitrap mass spectrometer (ThermoFisher Scientific).

The peptides were subjected to NSI source followed by tandem mass spectrometry (MS/MS) in Q ExactiveTM Plus (Thermo) coupled online to the UPLC. Intact peptides were detected in the Orbitrap at a resolution of 70,000. Peptides were selected for MS/MS using 28% NCE; ion fragments were detected in the Orbitrap at a resolution of 17,500. A data-dependent procedure that alternated between one MS scan followed by 10 MS/MS scans was applied for the top 10 precursor ions above a threshold ion count of 2E4 in the MS survey scan with 5.0s dynamic exclusion. The electrospray voltage applied was 2.0 kV. Automatic gain control (AGC) was used to prevent overfilling of the ion trap; 5E4 ions were accumulated for generation of MS/MS spectra. For MS scans, the m/z scan range was 350 to 1600 Da.

### 2.5. Database search

The resulting MS/MS data were processed using MaxQuant with integrated Andromeda search engine (v.1.4.1.2). Tandem mass spectra were searched against SwissProt_human database (20,274 sequences) concatenated with reverse decoy database. Trypsin/P was specified as cleavage enzyme allowing up to 2 missing cleavages, 4 modifications per peptide and 5 charges. Mass error was set to 10 ppm for precursor ions and 0.02 Da for fragment ions. Carbamido methylation on Cys was specified as fixed modification and oxidation on Met, Phosphorylation on Ser was specified as variable modification. False discovery rate (FDR) thresholds for protein, peptide and modification site were specified at 1%. Minimum peptide length was set at 7. All the other parameters in MaxQuant were set to default values.

### 2.6 Protein functional annotation

The Gene Ontology (GO) annotation proteome was root in the UniProt-GOA Database (http://www.ebi.ac.uk/GOA/). Proteins were categorized into biological process and molecular function using an in-house Perl script according to Gene Ontology (GO) terms. Kyoto Encyclopedia of Genes and Genomes (KEGG) were utilized to annotate pathways: firstly using KEGG online service tools KAAS to annotate proteins, secondly using KEGG online service tools KEGG mapper to map on the KEGG pathway database, finally using InterPro database and InterProScan to annotate protein domains and applying CORUM database to annotate protein complex.

### 2.7 Functional enrichment analysis

Fisher’s exact test was applied to test for enrichment or depletion (two-tailed test) of specific annotation terms among members of resulting protein clusters. The derived p-values were further adjusted to address multiple hypotheses by the method proposed by Benjamini and Hochberg. Any terms with adjusted p-values below 0.05 in any of the clusters were treated as significant.

### 2.8 Phosphorylated protein secondary structure analysis

The local secondary structures were predicted by NetSurfP method. The different secondary structure (alpha helix, beta strand and coil) probabilities of identified phosphorylated residues in this study were compared with the secondary structure probabilities at the position of control residues containing all Lys residues in our database. The distribution of phosphorylated and non-phosphorylated amino acids in protein secondary structures was analyzed.

### 2.9 Phosphorylated motif site analysis

The software motif-X was used to analyze enrichment or depletion of amino acids in specific positions of phos-13-mers (6 amino acids upstream and downstream of the phosphorylation site in all protein sequences. And all protein sequences in the database were used as background database parameter, other parameters with default.

### 2.10 Protein-protein interaction analysis

We analyzed the protein-protein interactions for the identified phosphorylated proteins using the Cytoscape software. The protein-protein interaction network was obtained from the STRING database, which defines a metric called the “confidence score”to define the interaction confidence; we fetched all interaction with a confidence score of at least 0.7 (high confidence).

### 2.11 Cell treatment and western blot analysis

For DNA damage treatment, separate populations of HeLa cells were seeded in equal cell numbers onto 150 mm dishes. The following day the cells were treated with thymidine (Sigma-Aldrich) at a concentration of 2.5 mM for approximately 14 h. For methyl methanesulfonate treatment, HeLa cells were simultaneously treated with MMS (Sigma-Aldrich) diluted to a final concentration of 0.05% during the final hour of thymidine treatment. For ultraviolet treatment, Hela cells were treated by 130mJ/cm2. Cells were then harvested for the acute time point. Collected cells were lysed with RIPA buffer containing protease inhibitor cocktails (Roche, Basel, CH). Samples were denatured, separated by SDS-PAGE, transferred onto a PVDF membrane, and incubated with primary antibody (1000×diluted, Abcam, Cambridge, GB) and secondary antibody (2000×diluted, Ptg lab, Chicago, USA) sequentially after blocking. The membrane was further washed with TBST and developed using ECL reagents (Pierce). For protein phosphorylation site validation, we mutated serine 38 in RPA1 and threonine 76 in RFC3 to alanine. Transfected cells were harvested and the supernatant of cell lysate were incubated with M2 beads to precipitated target protein. The phosphorylation level of each protein for both wildtype and mutant was detected by anti-serine phosphorylation or anti-threonine phosphoryltaion antibody and visualized by chemiluminiscence.

## 3. Results

### 3.1. Quantification of phosphorylated peptides and proteins in HeLa cells following UV treatment

**Fig 1:**
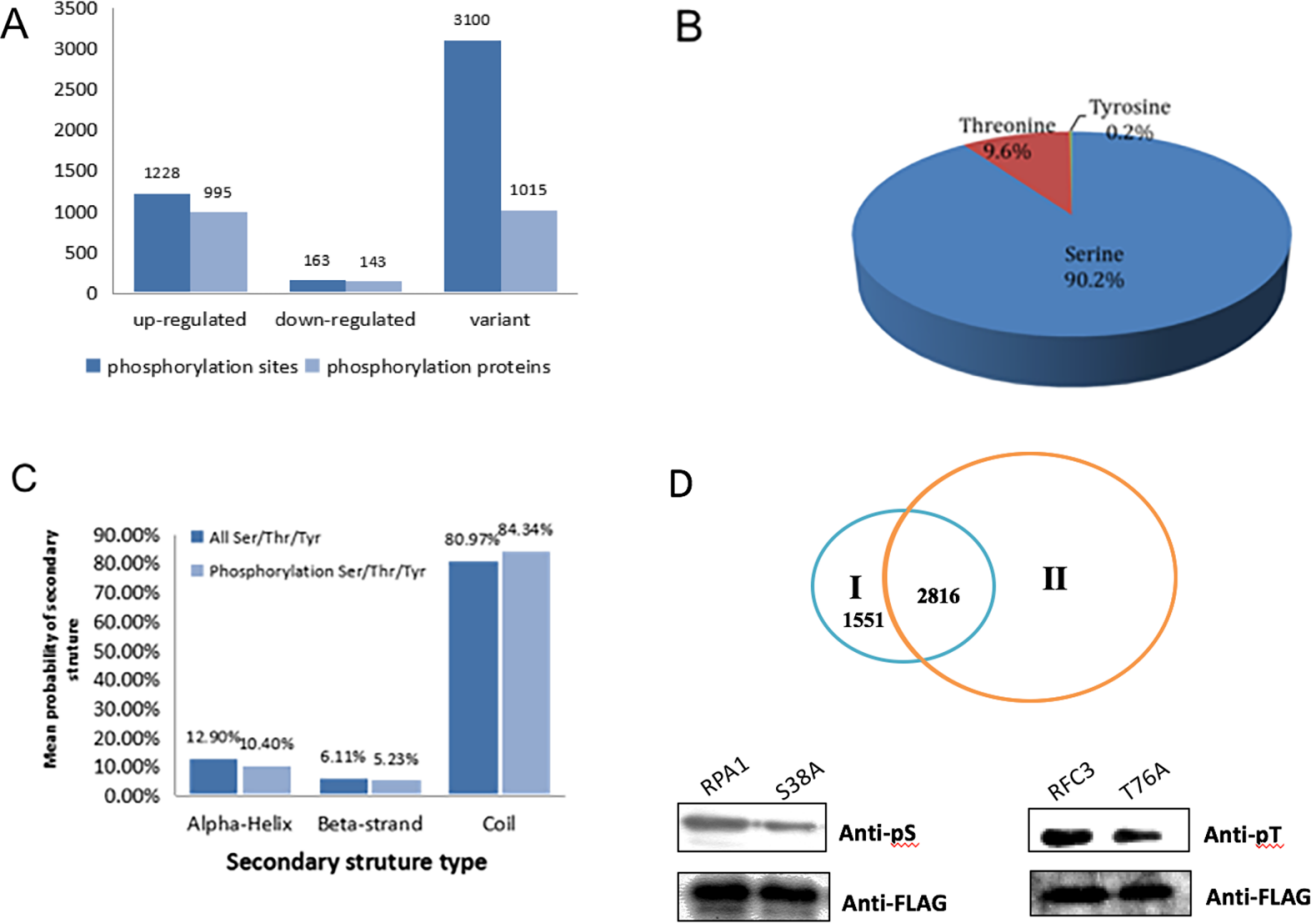
Quantitative overview of phosphorylated peptides and proteins in *HeLa* cells following UV treatment. (A) Number of phosphorylated proteins and proteins’sites quantified in HeLa cells in response to UV treatment. (B) Distribution of identified phosphorylated peptides at Serine, Threonine and Tyrosine sites. (C) Distribution of phosphorylated and non-phosphorylated amino acids in secondary structure. (D) Comparison of phosphorylated peptides identified in this study (I) and Phospho.ELM database (II) (http://phospho.elm.eu.org/).Validation of phosphorylation sites in RPA1 (S38) and RFC3 (T76) by western blot. Plasmids with 3xFLAG-S38A or 3xFLAG-T76A mutation were transfected to HeLa cells and precipitated by M2 beads. The phosphorylation level between wildtype and mutant proteins were evaluated by western blotting using specific antibody.

Reversible protein phosphorylation of serine, threonine and tyrosine are critical processes in prokaryote and eukaryote organisms [23]. After UV exposure, we identified 4491 phosphorylation sites in 2153 proteins based on mass spectrometry and 4367 phosphorylation sites in 2100 proteins were quantified (Fig 1A, S1 Table). The quantified phosphorylated proteins were considered to be up-regulated if quantitative ratio > 2. If quantitative ratio < 0.5, they were considered as down-regulated. We identified that 1228 phosphorylation sites in 995 proteins (47.4%) were up-regulated and 163 phosphorylation sites in 143 proteins (6.8%) were down-regulated (Fig 1A). There were 4052 phosphorylation sites modified in serine, 431 in tyrosine and eight in threonine (Fig 1B). We compared the distribution of phosphorylated and non-phosphorylated amino acids in secondary structure and compared phospho-Ser/Thr/Tyr sites with all the Ser/Thr/Tyr residues. Under normal conditions, phospho-Ser/Thr/Tyr was significantly enriched in coli structures, reaching 80.97%. After UV treatment, the ratio of phospho-Ser/Thr/Tyr in coli structures was even higher, indicating that Ser/Thr/Tyr around protein coli structures was very important for cell regular growth and DNA damage response (Fig 1C). Among them, 1551 phosphorylation sites had not been previously reported according to the phospho.ELM database (http://phospho.elm.eu.org/) (Fig 1D, S2 Table). We chose two newly identified phosphorylation sites in our study to further validate the result. Serine 38 in replication protein A1 (RPA1) and threonine 76 in replication factor C3 (RFC3) were mutated to alanine. Plasmids contain these mutations were transfected into HeLa cells and the phosphorylation level of these proteins were detected by western blot. The phosphorylation level of S38A and T76A were both down-regulated compared to wildtype proteins indicating these two sites were indeed the phosphorylation sites *in vivo*. (Fig 1D).

### 3.2 Identification of phosphorylated site motifs and domains in response to UV treatment

**Fig 2:**
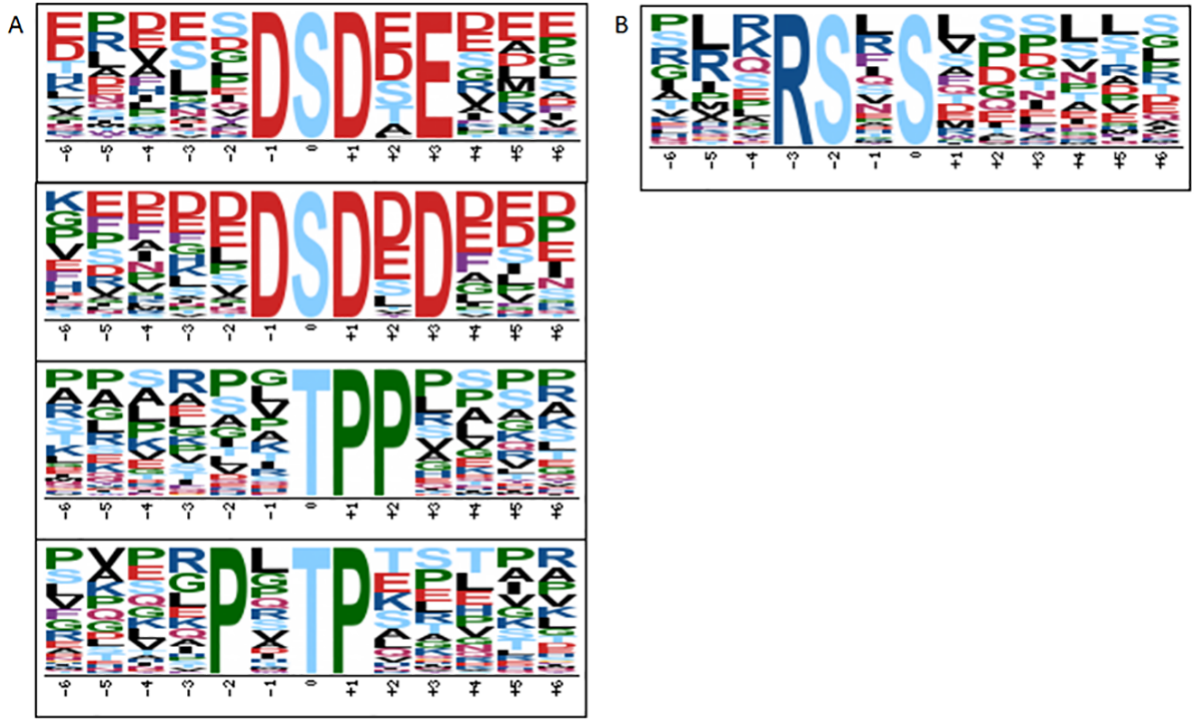
Phosphorylation-specific motifs using the Motif-X algorithm. (A) Pro-directed motif centered on Thr and Ser with a strong preference for additional Pro residues C-terminal to the phosphate. (B) Double-phosphorylation motifs found in our study.

To analyze the amino acid composition surrounding the identified phosphorylation sites, the frequencies of amino acids in 21 phosphorylation site-centered residues were analyzed. Pro, Asp, Glu and Ser appeared frequently in our data set. Pro often appeared at the ±1 position (following the phosphorylation site) after phospho-Ser or phospho-Thr. Besides Lys, Arg, Asp and Glu were appeared more likely surrounding Ser. Asp tended to be in ±1 positions and Arg in –2 or –3 locations. Only Pro and Arg were enriched following phospho-Tyr (Fig 2A). However, Arg often occurred when double-phosphorylation took place in two Sers (Fig 2B).

### 3.3 Subcellular localization and functional annotation of identified phosphoproteins under UV treatment

**Fig 3:**
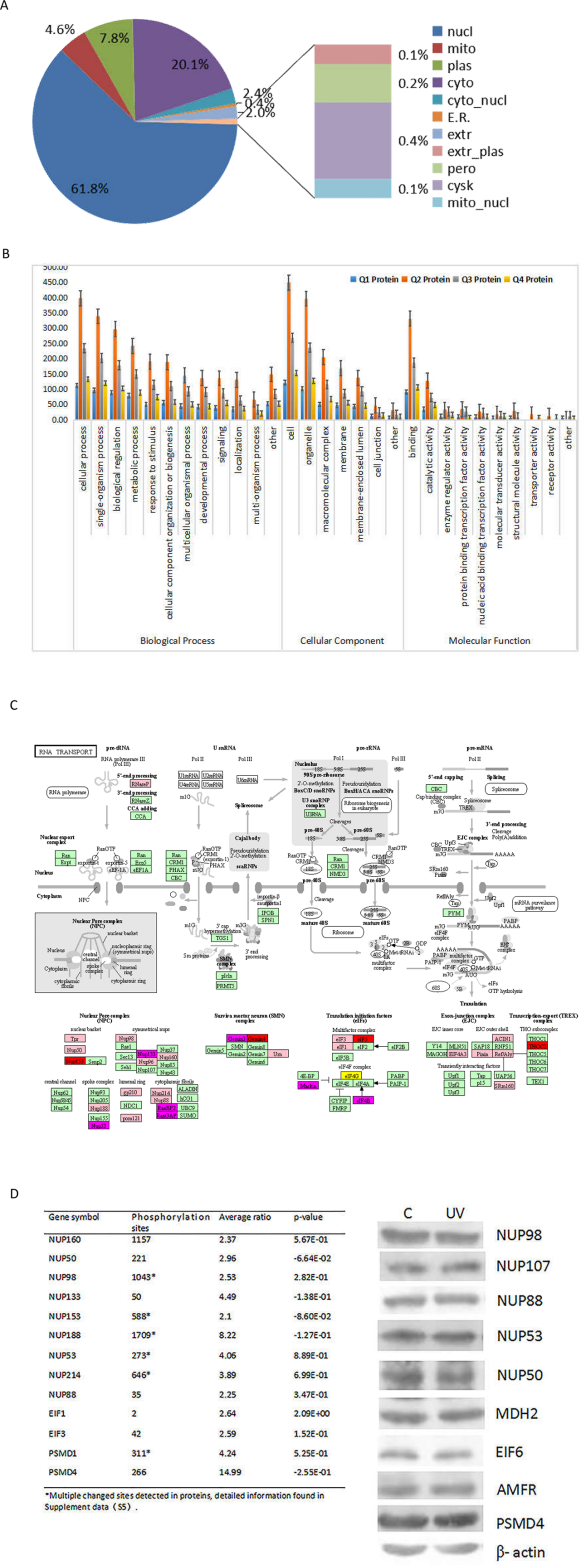
Subcellular localization and functional annotation of identified phosphoproteins under UV treatment. (A) The subcellular distribution of phosphorylated protein in HeLa cells under UV treatment. More than 80% of identified proteins were located in nucleus and cytoplasm. (B) Functional classification of identified proteins based on GO analysis and distribution of changed proteins in each pathway. Q1 presented down-regulated and Q2~Q4 presented different level of up-regulated. (C) Dynamic changes of phosphorylated proteins in RNA transport Pathway. Green represent ratio L/H<0.5. pink represent ratio L/H 2~3, amaranth represent ratio L/H 3~5, red represent ratio L/H>5.The phosphorylation level of nuclear pore complex protein such as Nup53, Nup133 and Nup153 were up-regulated, so does translation initiation factor proteins such as eIF1, eIF3 and eIF5. (D) Summarize of phosphorlated sites in RNA transport related proteins (left). Western blotting of total phosphorylation level of each protein before and after UV treatment (right). The phosphorylation level of detected proteins didn’t change significantly indicating phosphorylation at specific sites was more important in response to UV induced cellular stress.

To further explore the impact of differentially expressed proteins in cell physiological processes and discover internal relationships between differentially expressed proteins, we classified the functions of the differentially expressed proteins and analyzed the significance of functional enrichment including subcellular localization and GO annotation. As shown in Fig 3A, the nucleus contained 61.8% of phosphorylated proteins, 20.1% of the phosphorylated proteins occurred in cytoplasm and some occurred in other cell locations.

We further checked the modified proteins based on their molecular functions, cellular components and biological processes. Proteins involved in DNA binding and catalytic activity changed significantly after UV treatment, indicating that many nucleotide recruited binding proteins after phosphorylation in response to DNA damage stress. In cellular component, 102 proteins were down-regulated and 761 were up-regulated. There were 342 quantifiable proteins found in macromolecular complexes, and 51 of them were down-regulated. Membrane locations contained 284 related proteins and 48 of these were down-regulated. There were 74 proteins in cell junctions, 12 of them were down-regulated with phosphorylation. In biological process, the identified differentially expressed proteins included cellular progress (708), single-organism process (607), biological regulation (539), metabolic process (455) and response to stimulus (337) (Fig 3B, S3 Table).

KEGG pathway analysis found that the phosphorylation level of many proteins involved in different pathways was significantly changed after UV irradiation. For example, the phosphorylation level at specific sites of Raf-1, MEK, ERK and PKC in the RAP1 signaling pathway, FOXO and 14-3-3 in the PI3K-AKT signaling pathway, and Smad9 and ERK in the TGF-β signaling pathway were decreased after UV radiation (S4 Table). The phosphorylation level at specific sites of CHK1, CHK2 and cyclin B in the P53 signaling pathway; TRAF2, TAB and ELKS in the NF-kappa B signaling pathway and many proteins in purine and lipid metabolism greatly increased after UV radiation (S4 Table). It is noteworthy that many of the proteins listed above participated in different pathways and the phosphorylation level at different sites were changed in different ways, indicating multiple functions of these proteins and potential switches under certain conditions. In the other hand, the phosphorylation level of many proteins in nuclear pore complex, were changed significantly in response to UV irradiation, for example, the phosphorylation level at specific site(s) of NUP153, NUP133, NUP160 and NUP 88 were increased more than two fold under stress, while the phosphorylation level of site(s) in NUP37, NUP85, NUP155 and NUP93 were decreased significantly after treatment. So does translation initiation factor proteins such as eIF1, eIF3 and eIF5 which also increased the phosphorylation level at indicated site (Figs 3C, 3D and S5). We compared our data with those published in 2014 by Aaron *et al*. [24] who analyzed the dynamic changes in phosphorylated proteins following methyl methanesulfonate (MMS) treatment, and found that the modification pattern of nuclear pore complex-related proteins was quite different, indicating their different functions in response to different DNA damage types.

### 3.4 Phosphoprotein interactions and crosstalk under UV treatment

**Fig 4:**
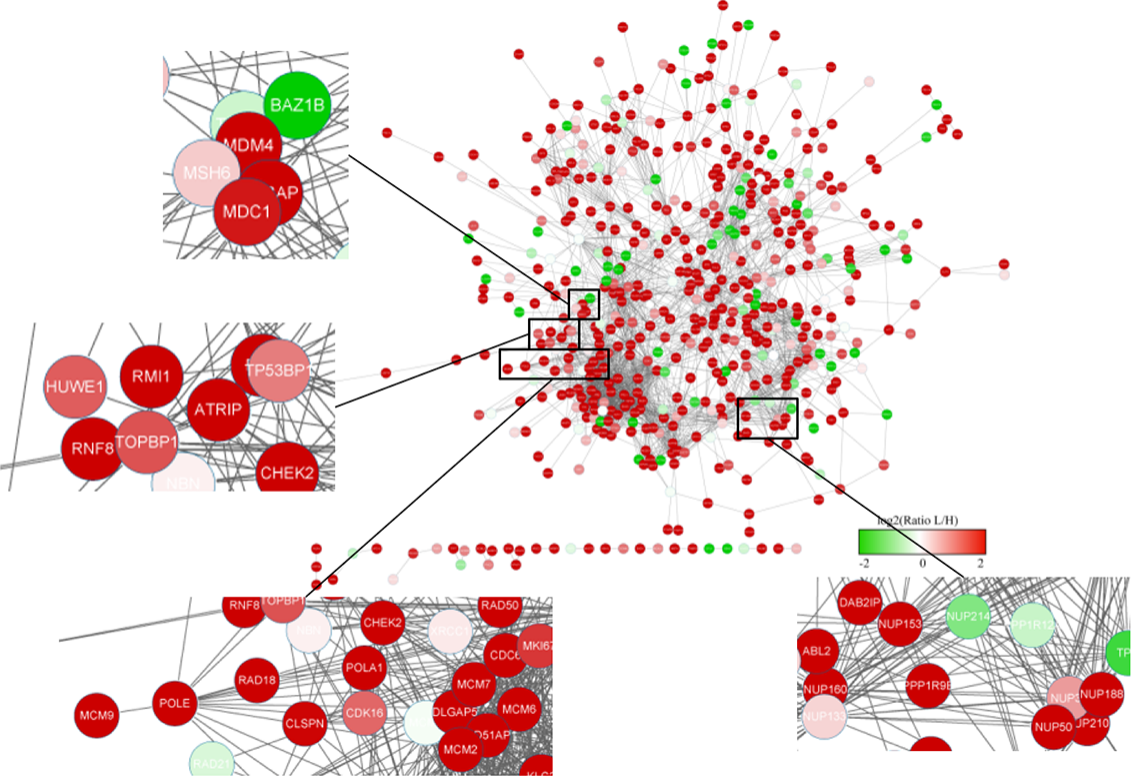
Network of phosphoproteins derived from data and the expanding view of phosphorylation level changes for parts of representative proteins. The different colors represent different ratios from -2 to +2. The highlight part are mismatch repair related protein—MSH6 network picture, DNA replication related protein—POLA1 and POLE network and nuclear pore complex protein—Nup153, Nup50, Nup188 and Nup214 network part.

Since the modification level change of a protein may result from a modification level change of another protein and may not be the direct cause of the cellular phenotype, additional information is required. Network-based analyses of protein–protein interaction (PPI) delineate the known associations among proteins in the context of biochemistry, signal transduction and biomolecular networks. We therefore aimed to integrate the interactome (i.e. PPI) to construct a global view of protein phosphorylation events under cellular DNA damage stress (Fig 4). In response to UV-induced DNA damage, the phosphorylation levels of proteins related to damage response and cell cycle progression such as CHEK2 and RNF8 were changed significantly. Meanwhile, the component proteins of the key genome replication complex (MCM) such as MCM4, MCM6 and MCM7 also showed different phosphorylation patterns after UV irradiation. The nuclear pore complex-related proteins, which were discussed above, also showed strong connections to responses to UV-induced DNA damage. The comprehensive analysis of phosphorylated proteins provided detailed clues for further investigation.

**Fig 5:**
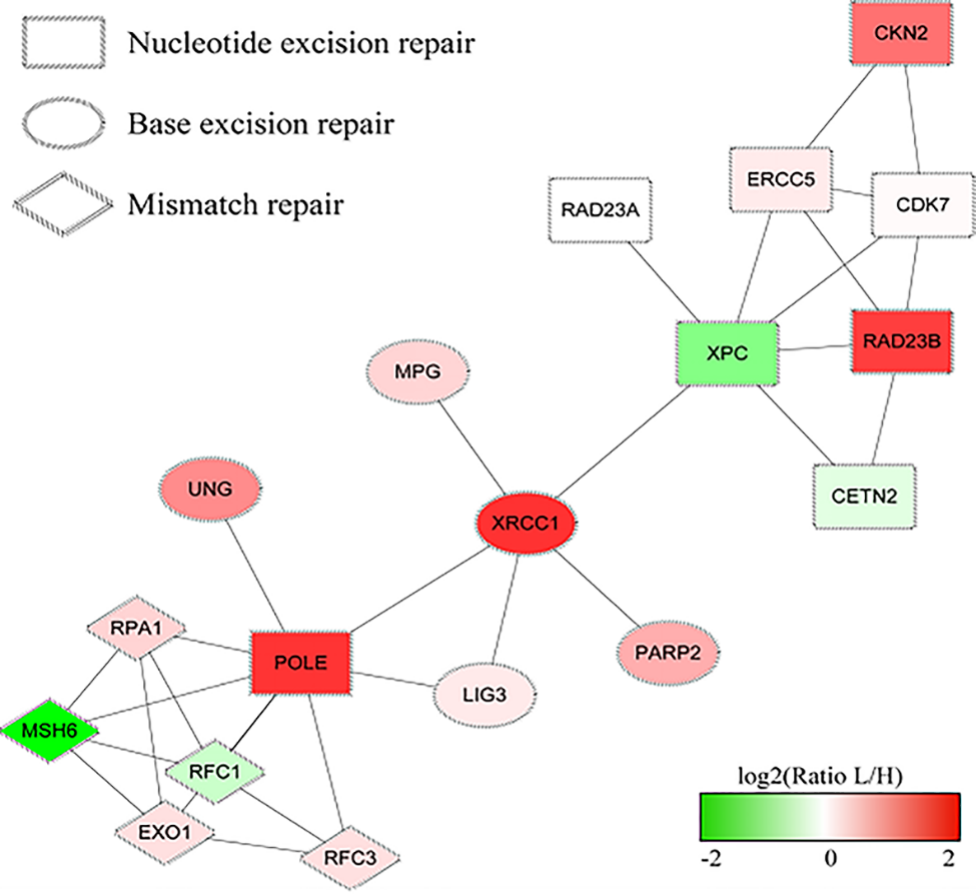
Influenced phosphoproteins inrelated to DNA repair pathway under UV treatment. Diamond represents mismatch repair proteins, ellipse represents base excision repair proteins and rectangle stands for nucleotide excision repair proteins. The different colors represent different ratios of phosphorylation level from -2 to +2.

To further focus on the dynamic changes in DNA repair proteins, we analyzed their modification levels and constructed a map (Fig 5). The phosphorylation of Ser261 on MSH6 decreased two-fold in response to UV treatment. MSH6 is thought to be an essential member in the mismatch repair pathway. It binds with MSH2 to activate an Mlh1-Pms1 endonuclease that works together with PCNA in exo1-independent mismatch repair. Phosphorylation of Ser266 on XRCC1 increased more than five-fold in response to UV treatment and was detected to interact with nucleotide excision repair protein XPC. This is an important protein function in the base excision repair pathway related to breast cancer. POLE, an important DNA polymerase closely associated with DNA repair and chromosomal DNA replication, is thought to interact with mismatch repair proteins, RPA1, RFC1, RFC3, and base excision repair proteins XRCC1[24],The phosphorylation level of this protein was also found increased in this work.

## 4. Discussion

Our research data showed single, double, triple- and even multiple-phosphorylated peptides, so our enrichment procedure was not biased by the degree of phosphorylation on peptides. Interestingly, many phosphorylated proteins had more than one phosphorylation site and some of these sites were very close to the next site, but to diverse degrees. For example, there were three phosphorylation sites in Zinc finger MYM-type protein 4, two of which were Ser1064 and Ser1071. Phosphorylated Ser1064 was up-regulated, while phosphorylated Ser1071 was down-regulated. Checking in the PDB database showed that these sites in proteins were not released publicly and so need further study.

Nuclear pore complexes mediate transport between the nucleus and cytoplasm and tether chromatin to create an environment for gene regulation. Previous study confirmed that depletion of specific nucleoporins could both positively and negatively affect DNA damage signaling, such as DNA damage response [25]. This is thought to regulate transcriptional activity of these regions [26]. In our study, the protein modification level of nuclear pore complex changed greatly after UV radiation, as also noted by Aslanian [26]. We concluded that this set of proteins was quite active under DNA damage treatment and phosphorylation at different sites can regulate different cellular processes to execute different function. To determine the detailed mechanism, a set of specific site phosphorylation antibodies will be necessary for further detection and analysis.

Since phosphorylation is a common PTM in organisms, many phosphorylated proteins contributed to a map of function crosstalk. In this map, many proteins involved in the DNA repair pathway interact with others and play important roles, such as MSH6 in the DNA mismatch repair pathway and XRCC1 in the base excision repair pathway. XRCC1 is a crucial component of the BER pathway. There have been many studies of single nucleotide polymorphism of XRCC1, especially of Arg399Gln and Arg194Trp. XRCC1 polymorphisms were thought to be closely related to many cancers, such as cervical cancer, non-small cell lung cancer and childhood acute lymphoblastic leukemia. In our study, we found a new site—Ser266 in the XRCC1 protein—that was related to DNA damage caused by UV exposure. More details are needed for dynamic analysis and the use of mass spectrometry-based proteomics will enable us to discover most UV-responsive downstream proteins and determine the functional crosstalk.

## Supporting information

S1 Table All quantified proteins based on mass spectrometry in our study. After UV exposure, we identified 4491 phosphorylation sites in 2153 proteins and 4367 phosphorylation sites in 2100 proteins were quantified, The quantified phosphorylated proteins were considered to be up-regulated if quantitative ratio > 2. If quantitative ratio < 0.5, they were considered as down-regulated.
(XLS)

S2 Table Comparison between our data and phospho.ELM database. There are 1551 phosphorylation sites in our study had not been previously reported according to the phospho.ELM database.
(XLSX)

S3 Table Functional enrichment-based clustering for expressed quantitation of protein modification. (XLSX)

S4 Table Phosphorylation level of many proteins involved in different pathways was significantly changed after UV irradiation. The phosphorylation level at specific sites of Raf-1, MEK, ERK and PKC in the RAP1 signaling pathway, FOXO and 14-3-3 in the PI3K-AKT signaling pathway, and Smad9 and ERK in the TGF-β signaling pathway were decreased after UV radiation. The phosphorylation level at specific sites of CHK1, CHK2 and cyclin B in the P53 signaling pathway; TRAF2, TAB and ELKS in the NF-kappa B signaling pathway and many proteins in purine and lipid metabolism greatly increased after UV radiation.
(XLSX)

S5 Table Phosphorylation level of many proteins in nuclear pore complex, were changed significantly in response to UV irradiation. The phosphorylation level at specific site(s) of NUP153, NUP133, NUP160 and NUP 88 were increased more than two fold under stress, while the phosphorylation level of site(s) in NUP37, NUP85, NUP155 and NUP93 were decreased significantly after treatment. So does translation initiation factor proteins such as eIF1, eIF3 and eIF5 which also increased the phosphorylation level at indicated site.
(XLSX)
